# Risk of target coverage loss for stereotactic body radiotherapy treatment of synchronous lung lesions via single‐isocenter volumetric modulated arc therapy

**DOI:** 10.1002/acm2.13145

**Published:** 2020-12-20

**Authors:** Lana C. Critchfield, Mark E. Bernard, Marcus E. Randall, Ronald C. McGarry, Damodar Pokhrel

**Affiliations:** ^1^ Medical Physics Graduate Program Department of Radiation Medicine University Kentucky Lexington KY USA

**Keywords:** BED10, coverage loss, lung SBRT, setup errors, single‐Isocenter VMAT, synchronous

## Abstract

Treating multiple lung lesions synchronously via single‐isocenter volumetric modulated arc therapy (VMAT) stereotactic body radiation therapy (SBRT) improves treatment efficiency and patient compliance. However, aligning multiple lung tumors accurately on single pretreatment cone beam CTs (CBCTs) can be problematic. Tumors misaligned could lead to target coverage loss. To quantify this potential target coverage loss due to small, clinically realistic setup errors, a novel simulation method was developed. This method was used on 26 previously treated patients with two metastatic lung lesions. Patients were treated with 4D CT‐based, highly conformal noncoplanar VMAT plans (clinical VMAT) with 6MV‐flattening filter free (FFF) beam using AcurosXB dose calculation algorithm with heterogeneity corrections. A single isocenter was placed approximately between the lesions to improve patient convenience and clinic workflow. Average isocenter to tumor distance was 5.9 cm. Prescription dose was 54 Gy/50 Gy in 3/5 fractions. For comparison, a plan summation (simulated VMAT) was executed utilizing randomly simulated, clinically relevant setup errors, obtained from pretreatment setup, per treatment fraction, in Eclipse treatment planning system for each of the six degrees of freedom within ± 5.0 mm and ± 2°. Simulations yielded average deviations of 27.4% (up to 72% loss) (*P* < 0.001) from planned target coverage when treating multiple lung lesions using a single‐isocenter plan. The largest deviations from planned coverage and desired biological effective dose (BED10, with α/β = 10 Gy) were seen for the smallest targets (<10 cc), some of which received < 100 Gy BED10. Patient misalignment resulted in substantial decrease in conformity and increase in the gradient index, violating major characteristics of SBRT. Statistically insignificant differences were seen for normal tissue dose. Although, clinical follow‐up of these patients is ongoing, the authors recommend an alternative treatment planning strategy to minimize the probability of a geometric miss when treating small lung lesions synchronously with single‐isocenter VMAT SBRT plans.

## 1 INTRODUCTION

Stereotactic body radiation therapy (SBRT) has become a standard of care for selected early‐stage nonsmall cell lung cancer (NSCLC) patients.^1^, ^2^, ^3^, ^4^ Furthermore, SBRT of solitary primary or metastatic lung lesions is a fast, safe, and effective treatment option with a high control rate comparable to surgery.^4^ For elderly medically inoperable patients, SBRT treatment has been shown to be effective.^5^ However, elderly patients or those with poor pulmonary function and multiple oligometastastic (<5 lesions) lung lesions may not retain their treatment position for long SBRT treatment times. Traditional SBRT treatment to lung lesions requires an individual plan for each lesion with a separate isocenter placed in each, prolonging patient setup and treatment time. Treating multiple lung lesions synchronously with a single‐isocenter plan, either using intensity‐modulated radiation therapy (IMRT) or volumetric arc therapy (VMAT), has been studied.^6^, ^7^, ^8^, ^9^ Single‐isocenter/multilesion VMAT lung SBRT treatments have been shown to be fast and efficient, improving patient comfort.^10^, ^11^, ^12^, ^13^, ^14^ Additionally, treatment efficiency and dose buildup at the tumor interface is improved with use of a flattening filter free (FFF) beam.^15^, ^16^, ^17^, ^18^ This faster treatment option could potentially reduce intrafraction motion errors and improve patient compliance.^10^

Despite the growing interest in single‐isocenter/multilesion VMAT lung SBRT treatments, there is a decrement in accuracy when treating multiple lesions synchronously compared to treating the lesions individually. When each lesion is treated separately, the treatment plan has an isocenter in the center of the lesion, and daily cone beam CT (CBCT) alignment corrections can be made focusing on that lesion. Single‐isocenter/multilesion VMAT plans are not robust against setup errors because one or all lesions could be offset from the single‐isocenter location, potentially resulting in less accuracy. Moreover, in many treatment planning systems (TPS) including Eclipse TPS (Varian Medical Systems, Palo Alto, CA, USA), there is no way to simulate residual setup errors in all six degrees of freedom (6DoF) without using “third party” software. Herein is described a simple and clinically useful method for demonstrating the dosimetric effects of setup errors in Eclipse TPS. To simulate and quantify possible treatment inaccuracy, this tool uses simulation CT images, identical beam data, and the original clinical treatment plan including dose calculation algorithm, without introducing additional sources of errors, thus only simulating the dosimetric effects of patient setup uncertainties. Utilizing this method, it is demonstrated that when treating multiple lung lesions with a single‐isocenter VMAT‐SBRT plan, small but clinically representative setup errors may result in unacceptable loss of target coverage and unintended dose to normal tissues.

This comparison was undertaken to quantify the dosimetric impact of residual setup errors on target coverage and collateral dose to adjacent organs at risk (OAR) for single‐isocenter VMAT SBRT treatments of multiple lung lesions. Lung SBRT literature suggests that a biological effective dose (BED10) of ≥100 Gy (with α/β = 10 Gy) to each lesion is required for optimal tumor local control (LC) and overall survival.^19^, ^20^ Olsen and colleagues reported clinical outcomes of 130 lung SBRT patients treated with three different dosing schemes. They demonstrated that lung SBRT treatments to 45 Gy in 5 fractions (85.5 Gy BED10) provided inferior tumor LC rate (50% LC at 2 years) compared to 50 Gy in 5 fractions (100% LC at 2 years) and 54 Gy in 3 fractions (91% LC at 2 years), suggesting that at least 100 Gy BED10 is necessary. Thus, in addition to evaluating the loss of target coverage, this simulation study compared planned vs simulated BED10 to each lesion.

## 2 MATERIALS AND METHODS

After obtaining Institutional Review Board approval, 26 patients with two synchronous lung tumors who underwent single‐isocenter VMAT lung SBRT treatment of 54 Gy in 3 fractions or 50 Gy in 5 fractions were included in this study.

### 2.1 Patient setup and contouring

Patients were immobilized using the Body Pro‐Lok^TM^ SBRT system (CIVCO, Orange City, IA) in the supine position with arms up. A free‐breathing CT was obtained on a GE Lightspeed 16 slice CT scanner (General Electric Medical Systems, Waukesha, WI) with 512 × 512 pixel image size and 2.5 mm slice thickness in the axial helical mode. Respiratory assessment and motion management included abdominal compression (21 patients) or a 4D CT scan (5 patients) utilizing Varian RPM system (version 1.7). The 3D CT scan was brought into Eclipse Treatment Planning System (Version 15.6, Varian Medical Systems, Palo Alto, CA, USA). Both gross tumor volumes (GTVs) were contoured on the 3D CT. If a 4D CT was obtained, an internal target volume (ITV) was contoured based on the registered 4D CT reconstructed maximum intensity projection (MIP). The planning target volumes (PTVs) were created either by expanding a uniform margin of 5 mm from the ITV or in the case of no 4D CT, 5 mm expansion of the GTV in the lateral direction and 10 mm expansion in the superior‐to‐inferior direction. The target names (PTV1 or PTV2) were arbitrarily chosen by the treating physician. All planning was completed on the free‐breathing CT and Hounsfield units (HU) within the PTV were maintained per the planning CT dataset following our in‐house SBRT protocol. Average PTV size was 20.4 ± 16.2 cc (4.7–80.9 cc). Distance to isocenter was determined by finding the coordinates of the PTV geometric center and calculating Euclidian distance in 3D geometry with the isocenter coordinates. Critical structures were contoured including lungs (right, left, and combined), cord, heart, bronchus, trachea, esophagus, skin, and ribs (right, left, and combined). Tumor characteristics for the cohort are summarized in Table 1.

**Table 1:** Main tumor characteristics of the 26 lung SBRT patients included in this study. Each patient had 2 tumors.

Parameters	Mean ± STD (range or n = no. of patients)
Tumor 1, PTV1 (cc)	22.0 ± 19.7 (5.0–80.9)
Tumor 2, PTV2 (cc)	17.5 ± 11.6 (4.7–43.6)
Prescribed dose to each lesion	54 Gy in 3 fractions (n = 7)
	50 Gy in 5 fractions (n = 19)
Isocenter to tumor distance (cm)	5.9 ± 2.5 (range: 2.1–11.5)
Tumor location (left/right/bilateral)	(n = 7 / 7 / 12)
Uninvolved lung (cc) = lungs minus both PTV	3696.4 ± 1059.7 (1921.6–6785.6)

STD, standard deviation.

### 2.2 Clinical single‐isocenter VMAT plans

For all 26 patients, single‐isocenter VMAT lung SBRT plans were generated in Eclipse TPS for treatment on a Truebeam Linac (Varian Medical Systems, Palo Alto, CA, USA) consisting of standard millennium 120 MLC and 6 MV‐FFF (1400 MU/min) beam. A single isocenter was placed approximately between the two tumors. Doses were 54 Gy or 50 Gy in 3 or 5 fractions, respectively. Both PTVs (PTV 1 and PTV 2) were planned with dose prescribed to the 80% isodose line and optimized such that 95% of each PTV received 100% of the prescription dose. The maximum dose to the PTV fell inside the GTV. Full arcs (coplanar) were utilized for bilateral lung tumors and partial noncoplanar arcs utilized for uni‐lateral lung tumors, with ±5°–10° couch rotations, if possible. Optimal collimator angles and jaw tracking were chosen to reduce MLC leakage between each arc. Dose was calculated using the Boltzmann transport based AcurosXB algorithm for heterogeneity corrections with dose to medium reporting mode.^21^, ^22^, ^23^ Planning objectives followed RTOG guidelines.^24^, ^25^ Each of the clinical VMAT plans were delivered every other day to the patient in the clinic.

### 2.3 Simulated single‐isocenter VMAT plans

**Fig. 1:**
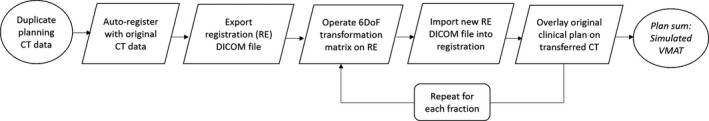
The workflow describes the steps required to complete simulation of isocenter misalignment in Eclipse TPS in all six dimensions. It utilizes image registration and the external beam treatment planning modules in Eclipse. The result is a plan summation of all treatment fractions that have been individually and randomly transformed, representing a clinically realistic treatment scenario.

**Fig. 2:**
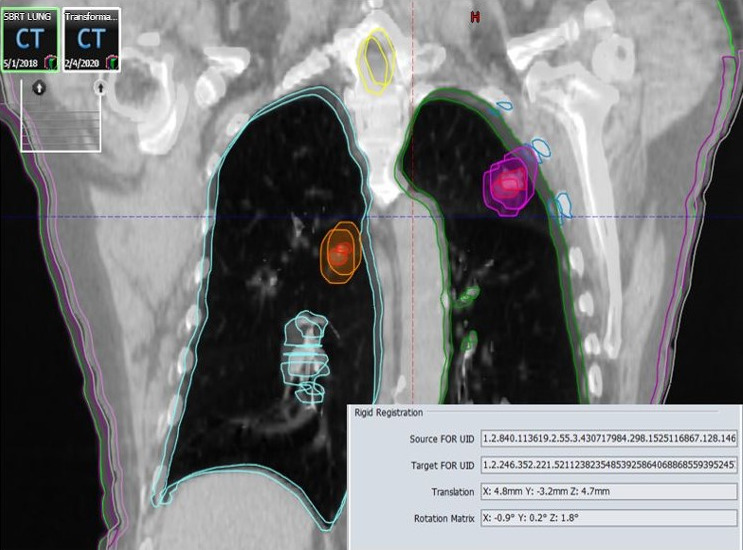
Above is a demonstration of a randomly rotated (within, ±2°) and translated (within, ±5 mm) CT data set (see bottom right inset) around the plan isocenter location (cross‐hair) for a representative patient (one fraction). The PTVs are shown in orange and pink and the GTVs are in red in both lungs. Normal structures are shown: lungs (light blue and green), skin (purple), cord (yellow), and ribs (blue). This patient was treated for 50 Gy in 5 fractions to both tumors, thus the random transformation process was repeated for a total of five treatments (see top left inset).

To evaluate patient setup uncertainties, clinically observable setup errors in all 6DoF were simulated in Eclipse TPS. Evaluation of pretreatment CBCT scans for our previously treated single‐isocenter VMAT treatments for thoracic lesions allowed for determination of clinically representative random interfraction setup errors to be within ±5 mm in the x‐, y‐, and z‐direction and within ±2° for pitch, yaw, and roll. The translational errors were defined for isocenter displacements. The rotational errors were defined for patient rotations relative to the isocenter around the right–left (pitch), anterior–posterior (yaw), and superior–inferior (roll) directions. For single‐isocenter VMAT treatment, our current clinical practice is that, if we observed these setup errors larger than ±5 mm in any translational and ±2° in any rotational direction, we re‐setup the patient and reimage for better alignment. Since demonstrating the loss of target coverage due to setup errors in current Eclipse TPS in all 6DoF was not readily accessible, an in‐house MATLAB (Math Works, MA, USA) script was written. This simulation method was developed and integrated into Eclipse TPS in order to achieve the desired transformations and recompute the simulated VMAT plan for each fraction. To reproduce the interfraction setup errors, this script allowed the boundary conditions to confine the randomly generated setup uncertainties within ±5 mm in each translational direction and ±2° in each rotational direction with respect to the single‐isocenter location as described above. The in‐house script utilizes a RE DICOM file that is created with an image registration in Eclipse. This RE DICOM file consists of the patient CT registered to itself, thus the transformation matrix between the two images is null. The MATLAB script utilizes a random number generator to rewrite the transformation matrix of one of the identical patient CT datasets to apply translations and rotations within the determined range of possible shifts. The random number generator utilized creates uniformly distributed random numbers, thus the transformation could simulate the worst‐case scenario for patient setup errors. The image registration workspace in Eclipse TPS allows for visualization of these rigid transformations in all 6DoF. This is repeated for the number of fractions, with the original plan copied to the transformed image. The result of the simulation process is a plan summation of all three or five randomly transformed treatment fractions that mimics day‐to‐day clinical scenarios, allowing for evaluation of a clinically representative single‐isocenter/multitumor VMAT lung SBRT treatment. Figure 1 below demonstrates the steps taken to achieve a complete simulated VMAT plan. Figure 2 demonstrates randomly transformed CT images, used for one treatment (out of five fractions) of a representative plan.

### 2.4 Plan comparison

All plans were compared per RTOG guidelines for target coverage along with maximum and volumetric dose to the adjacent OAR. Normal tissues that were evaluated included maximum dose to 0.03 cc of ribs, spinal cord, heart, bronchial tree, esophagus, and skin. Lung doses were evaluated using the mean lung dose (MLD), percentage of lung receiving 10 Gy (V10Gy) and 20 Gy (V20Gy) or more. Distance to isocenter was determined by utilizing the coordinates of the geometric center of each PTV as described above. In addition to the OAR doses, both plans were rigorously evaluated using the following metrics:

- Plan maximum dose: Maximum dose in the target
- Conformity Index (CI): Ratio of prescription isodose volume to the PTV volume. Values between 1.0 and 1.2 are desirable, but values between 1.2 and 1.5 would be acceptable per protocol with minor deviations. Therefore, for prescription isodose volume (V_RI_),

(1) $${\text{CI}}_{\text{RTOG}}=\frac{V_{\text{RI}}}{\text{PTV}}.$$

- Paddick Conformation Number (PCN): Determines the overlap of the prescription isodose volume and the PTV volume. Ideally, PCN = 1.0. For target volume covered by the prescription dose (PIV), and total volume covered by the prescription isodose volume, V_RI_,

(2) $${\text{PCN}}_{\text{Paddick}}=\frac{{\text{PTV}}_{\text{PIV}}^{2}}{\left(\text{PTV}\times V_{\text{RI}}\right)}.$$

- Heterogeneity Index (HI): Evaluates the dose heterogeneity inside the PTV,

(3) $$\text{HI}=\frac{D_{\text{Max}}}{\text{Rx}}.$$

- Gradient Index (GI): Used to evaluate the intermediate dose fall off,

(4) $$\text{GI}=\frac{50\%\text{Iso dose Volume}}{\text{PIV}}.$$

- Maximum dose at 2 cm away from the PTV in any direction (D2cm): Acceptable values depend on PTV size.
- Biological Effective Dose (BED10): For each PTV, BED10 was calculated using the prescribed dose to PTV D95% (Gy). For each GTV, BED10 was determined using the minimum dose (d) per fraction to the GTV. An α/β ratio of 10 Gy was used for the pulmonary tumor and for n = number of treatments, the BED10 was calculated using the following formula:

(5) $$\text{BED}10=n\times d\left[1+\frac{d}{\alpha /\beta }\right].$$

### 2.5 Statistical analysis

Data were assessed for normality, then either a paired two‐tail Student’s t‐test (Microsoft Excel, Microsoft Corp., Redmond, WA) or a Mann–Whitney test (Minitab, Minitab LLC, Chicago, IL, USA) was used to compare the data for the clinical VMAT vs simulated VMAT plans for all parameters of target coverage and dose tolerances to OAR. A value of *P* < 0.05 was considered statistically significant.

## 3 RESULTS

After simulation of setup errors, all PTVs had loss of dose coverage as well as some ITVs and GTVs. Simulated VMAT plans demonstrated an average PTV coverage loss of 27.4 ± 14.6%, with a maximum loss of 71.7% compared to the original clinical plans. Table 2 shows the analysis of target coverage for all 52 lesions. After the random transformations were applied, statistically significant decreases in PTV dose coverage, CI, PCN, and HI were observed. The drastic decrease in average CI and PCN for the simulated plans suggests that the prescription isodose volume was not covering the PTV as originally intended. It is important to note that for one patient the proximity of the lesions resulted in high and intermediate dose bridging between the lesions and thus a large CI of 2.69 was observed, which was reduced to 1.33 for the simulated plan. The GI increased from 5.24 ± 1.21 (3.66–8.31) for the original plans to 8.38 ± 3.78 (3.89–23.76) for the simulated plans. This suggests that due to small rotational and translational setup errors, there was significantly higher intermediate dose spillage, and the sharp dose fall off indicative of lung SBRT treatments no longer existed. For the smaller (<10 cc) target sizes, clinically unacceptable GI up to 23.76 was observed (see Table 2).

**Table 2:** Analysis of the dosimetric and delivery parameters for 26 lung SBRT patients treated with a single‐isocenter/multiple‐target VMAT plan.

Target	Parameter	Clinical VMAT	Simulated VMAT	*P*‐value
	Max target dose (%)	122.5 ± 3.8 (115.4–131.1)	121.6 ± 3.1 (115.3–128.6)	n. s.
PTV (n = 52)	% Volume covered by Rx dose (%)	96.1 ± 1.2 (95.0–98.8)	68.7 ± 14.7 (25.6–95.2)	**<0.001**
	CI	1.08 ± 0.25 (0.95–2.69)	0.75 ± 0.19 (0.26–1.33)	**<0.001**
	PCN	0.89 ± 0.03 (0.81–0.98)	0.64 ± 0.13 (0.26–0.85)	**<0.001**
	HI	1.21 ± 0.04 (1.13–1.31)	1.20 ± .04 (1.12–1.29)	**0.03**
	GI	5.37 ± 0.94 (3.66–7.2)	8.36 ± 3.7 (3.89–23.76)	**<0.001**
	D2cm (%)	51.7 ± 5.6 (38.8–67.0)	51.7 ± 5.1 (42.4–62.3)	n. s.
GTV (n = 52)	% Volume covered by Rx dose (%)	100 ± 0	99.4 ± 2.2 (87.7–100.0)	**0.02**
ITV (n = 10)	% Volume covered by Rx dose (%)	100 ± 0	99.3 ± 1.3 (95.7–100.0)	n. s.

Mean ± STD (range) and *P*‐values were reported for clinical VMAT and simulated plans. n. s., not significant; Significant values are highlighted in bold; STD, standard deviation; PCN, Paddick Conformation Number; n, no. of targets.

For all 52 lesions, the average GTV coverage loss following the random transformations was 0.6%. However, for PTV volumes less than 10 cc, the GTV coverage loss was the greatest at an average of 1.6%, with a maximum loss of up to 12.3% in some cases. For the subset of five patients with an ITV (n = 10) contours, statistically insignificant ITV coverage loss following the random transformation was observed (<1%, on average), however, in some cases the loss of ITV coverage was up to 4.3%.

**Fig. 3:**
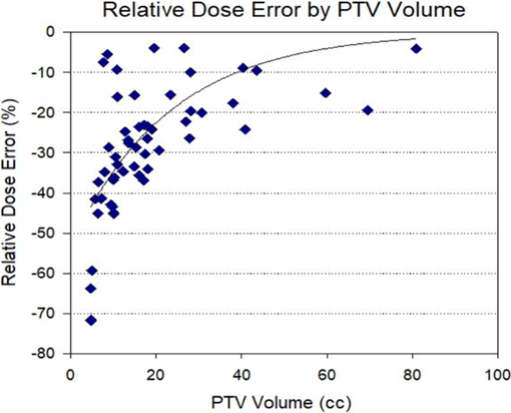
Loss of target coverage for all 52 lesions plotted as a function of PTV size. The curve fit (R^2^ = 0.43) indicates a probable correlation between the target size and coverage loss, suggesting that setup errors will result in a larger coverage loss for small PTV sizes.

**Fig. 4:**
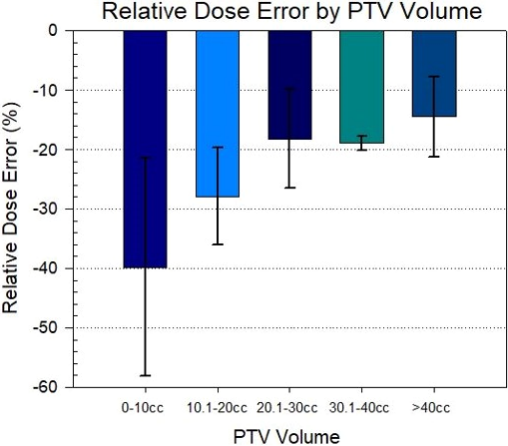
Loss of target coverage for all 52 lesions as a function of binned PTV sizes. The largest coverage loss was seen for lesions less than 10 cc. Target sizes ≤ 10 cc exhibited average coverage losses of 40%, up to 70% in some cases. The corresponding GTV loss for this subset was an average of 1.6%, up to 12.3% in some cases.

**Fig. 5:**
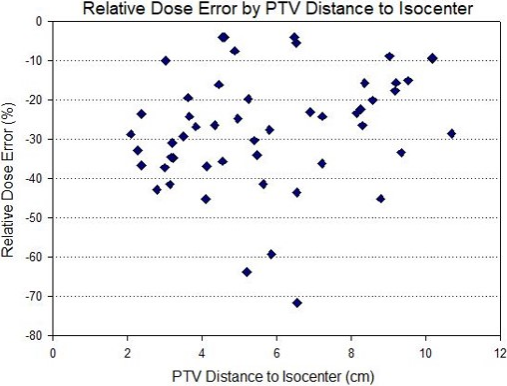
Scatter plot of relative dose error for all 52 lesions as a function of distance to isocenter. For randomly assigned rotational (±2°) and translations (±5 mm) errors in each direction, no clear relationship between the loss of target coverage and distance to isocenter was observed suggesting that random translational shifts dominated the loss of target coverage. However, the largest coverage loss (>50%) was seen for those lesions with the smallest PTV of about 5 cc.

**Fig. 6:**
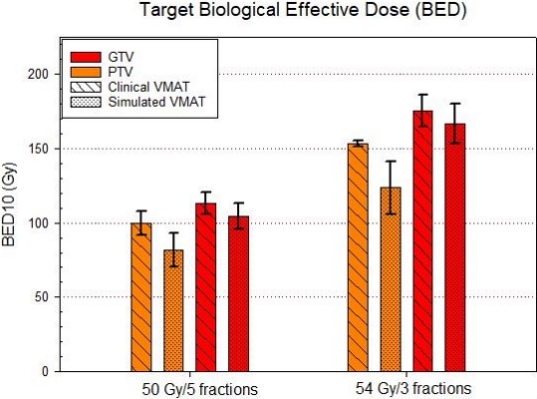
Comparison of calculated BED10 for both PTV and GTV for all 52 targets is shown for both plans. For patients treated with 54 Gy in 3 fractions, >100 Gy BED10 was always preserved for each PTV and GTV even with residual setup errors. However, for patients treated with 50 Gy in 5 fractions (see left panel), the average BED10 for PTV and GTV were 81.5 Gy and 104.4 Gy, respectively, with simulated VMAT plans compared to 100.0 Gy and 113.0 Gy BED10 with original VMAT plans, suggesting that there is a risk of under dosing targets due to setup errors.

The greatest target coverage loss was seen with the smallest PTV sizes. For PTV volumes less than 10 cc (n = 14), the relative dose error for the simulated VMAT plans was −39.8 ± 18.3% with respect to the clinical VMAT plans. For PTV volumes greater than 10 cc (n = 38), the average relative dose error was −19.8 ± 6.1%. Figure 3 demonstrates the trend in relative dose error with respect to PTV sizes while the relative dose error binned by PTV volume is shown in Fig. 4. However, no obvious correlation between the PTV coverage loss and distance to isocenter was observed, as shown in Fig. 5. It indicates that random translational shifts dominated the loss of target coverage in these simulations. However, the largest coverage loss with greater than 50% was observed for those lesions with the smallest target size of about 5.0 cc. Figure 6 demonstrates BED10 calculated utilizing the minimum dose received by the GTV, whereas the BED10 for the PTV was calculated using the dose covering 95% of the PTV volume as described above. For the 54 Gy in 3 fractions dosing scheme, it always maintained a high BED10 (>100 Gy) to the both PTV and GTV even with these clinically observable simulated setup errors for a single‐isocenter/multiple lesions suggesting that there was no underdosing of target(s). However, for all patients receiving 50 Gy in 5 fractions, any loss in PTV coverage resulted in a BED10 < 100 Gy (*P* < 0.001) as shown in left panel of Fig. 6. For one patient, the BED10 for a GTV was only 83.0 Gy suggesting suboptimal SBRT treatment to that patient. Although, for other cases the GTV BED10 was still >100 Gy, signifying the acceptable tumor local control is possible.

Table 3 shows the comparisons of maximum doses to normal tissues. The maximum dose to the skin, ribs, and esophagus were all lower for the simulated VMAT plans and were statistically significant, however, likely not clinically significant. The largest decrease in maximum ribs dose for one patient was 10.9 Gy in which case both lesions (PTV1, 5.0 cc and PTV2, 16.1 cc) were proximal to the chest wall. Despite the loss of target coverage for the PTVs demonstrated in Table 2, the uninvolved lung V20Gy, V10Gy, and MLD did not change significantly suggesting that the doses intended for the PTVs were not subsequently deposited in the uninvolved lungs.

**Table 3:** Analysis of the maximal dose to OAR for 26 lung SBRT patients.

	Parameter	Clinical VMAT	Simulated VMAT	*P*‐value
Maximal dose to OAR and uninvolved lung	Skin (Gy)	17.7 ± 3.6 (11.0 – 26.6)	16.8 ± 3.5 (10.4–25.8)	**<0.001**
	Ribs (Gy)	45.2 ± 11.2 (22.5 – 59.0)	42.8 ± 9.2 (24.7–59.1)	**0.003**
	Spinal cord (Gy)	10.5 ± 3.3 (4.7 – 15.5)	10.6 ± 3.5 (4.5–16.1)	n. s.
	Heart/Pericardium (Gy)	21.1 ± 11.5 (0.9 – 52.0)	20.5 ± 11.8 (0.9–54.2)	n. s.
	Bronchus (Gy)	18.2 ± 11.9 (0.8 – 50.4)	17.9 ± 12.4 (0.7–51.1)	n. s.
	Esophagus (Gy)	16.2 ± 8.2 (5.7 – 43.5)	15.8 ± 8.1 (5.6–41.9)	**0.005**
	V20Gy (%)	6.8 ± 4.1 (2.1 – 17.0)	6.9 ± 4.1 (2.0–17.5)	n. s.
	V10Gy (%)	18.3 ± 10.1 (6.8 – 43.6)	18.5 ± 10.2 (6.8 – 44.0)	n. s.
	MLD (Gy)	5.6 ± 2.5 (2.4 – 10.9)	5.5 ± 2.5 (2.3 – 10.9)	n. s.

Mean ± STD (range) and *P*‐values were reported for clinical VMAT and simulated VMAT plans. n. s., not significant; Significant values are highlighted in bold. STD, standard deviation.

**Fig. 7:**
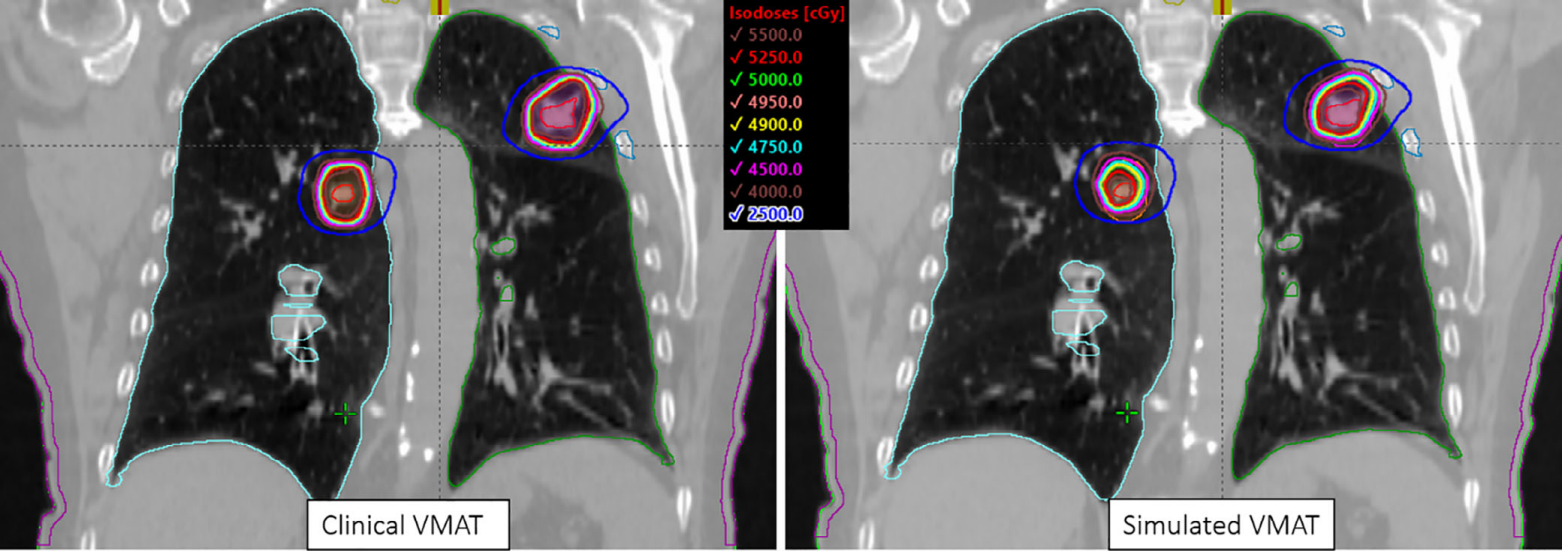
Coronal view of the isodose distribution is shown for the clinical VMAT plan (left panel) and the simulated VMAT plan (right panel), showing the significant loss of target coverage (see higher isodose lines with respect to both PTVs). The single‐isocenter location is shown by the cross‐hair. For this patient, the bronchus dose increased by 3.7 Gy with simulated VMAT whereas the maximal rib dose was decreased by 5.8 Gy.

**Fig. 8:**
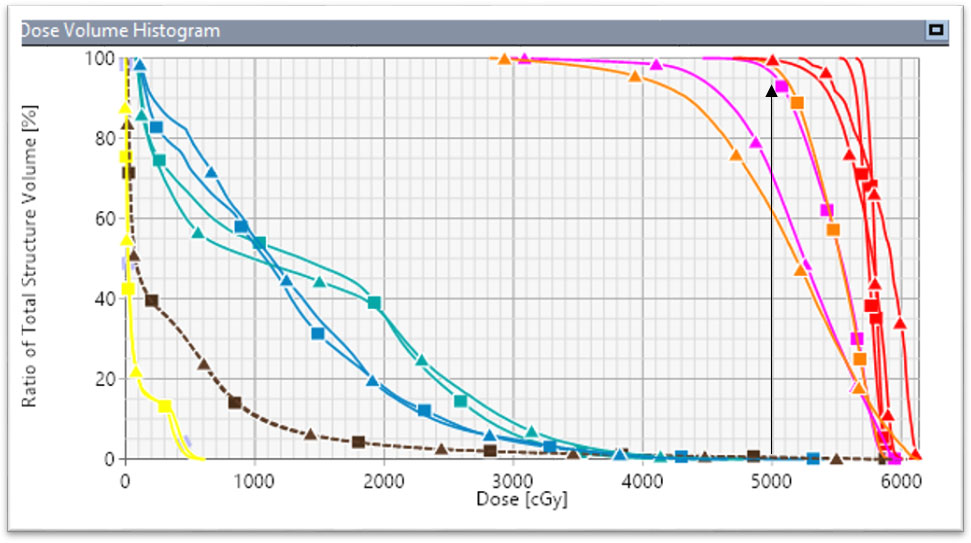
Dose volume histogram for the example patient shown. The vertical black arrow shows the original planned coverage for both PTV1 (orange) and PTV2 (pink). Squares represent the clinical VMAT plan and triangles represent the simulated VMAT plan. Shown are the GTVs (red), total lung (brown), ribs (light blue), bronchus (cyan), and spinal cord (yellow). The right PTV1 (6.5 cc) and the left PTV2 (18.0 cc) lost 37.2% and 26.5% coverage, respectively, compared to the original clinical 95% PTV coverage of both targets. A small loss of GTV coverage was seen with the simulated VMAT plan.

Dose to the bronchus did not change significantly between clinical VMAT and simulated VMAT plans. However, the largest increase in maximal dose to bronchus was 3.7 Gy for the example patient shown in Fig. 7, although still acceptable per RTOG‐0813 protocol.^25^ Figure 8 shows the DVH associated with this patient.

## 4 DISCUSSION

A novel and clinically useful tool was developed to simulate and quantify the dosimetric effects of interfraction setup errors in the case of synchronous multiple lesions treated with a single‐isocenter VMAT lung SBRT plan. After applying random translational shifts of ±5 mm and rotational errors of ±2° in each direction, dramatic loss of PTV coverage was observed with an average relative dose error of 27.4 ± 14.6% (up to 71.7% in some cases) (*P* < 0.001). Smaller tumors (<10 cc) exhibited the greatest PTV coverage loss with random rotations and translations at 39.8 ± 18.3% and 19.8 ± 6.1% for <10 cc and ≥10 cc, respectively. Overall, the GTV dose error was less than 1%, on average, but for smaller target sizes was up to −12.3% (*P* = 0.02). Major dosimetric differences were observed in loss of target conformity (*P* < 0.001) and gradient index (*P* < 0.001), negatively affecting the steep dose gradient desired in SBRT treatments. The change in dose to most normal tissues was statistically insignificant and probably clinically unimportant, unless critical structures are abutting the target or if re‐irradiation is being considered. In this 6DoF simulation, there was no clear trend of PTV coverage loss as a function of distance to isocenter. This is likely due to changing depths and SSD affecting the dose calculation rather than the dose just being shifted with respect to the patient’s anatomy. Also effecting this trend are randomly generated clinically realistic translational errors of ±5 mm (in each direction) dominating the small but clinically observed rotational error of ±2° (in each direction) or could be due to the vast array of PTV sizes obscuring the coverage loss. This is consistent with previously published spine SBRT treatment data.^26^ For instance, Wang et al from MD Anderson Cancer Center demonstrated that dosimetric effects from isocenter translational displacement of 1–3 mm were more severe than that from patient rotations of 1–3°. Although their study has shown the results of setup uncertainties in the case of a single spine lesion treated with an isocenter at the center of the target, a 2‐mm translational setup error resulted in clinically significant loss of target coverage.

Using a total of 124 patients with 159 pulmonary lesions treated with variable fractionation schemes of SBRT, Guckenberger *et al* ^19^ demonstrated that doses of greater than 100 Gy BED10 to the CTV based on 4D CT dose calculation resulted in excellent tumor local control rates (90% at 3 years). Their CTV was generated in the CT pulmonary window and the ITV was the sum of the CTV positions in inhalation and exhalation, similar to our GTV volume. In this study, the BED10 for the GTV was calculated utilizing the minimum dose received by the GTV, whereas the BED10 for the PTV was calculated using the dose covering 95% of the PTV volume as described above. We have demonstrated that due to residual setup errors, for all patients receiving 50 Gy in 5 fractions, any loss in PTV coverage resulted in a BED10 <100 Gy (*P* < 0.001) (see Fig. 6, left panel). For one patient, the BED10 for a GTV was only 83.0 Gy. However, for the majority of cases the GTV BED10 was still >100 Gy, suggesting that acceptable tumor local control is likely. For the five patients who had an ITV, all received a prescription dose of 50 Gy in 5 fractions. For these patients, three had ITV which received a minimum dose less than 50 Gy and thus did not achieve a BED10 of >100 Gy. In the case of the low BED10, whether 5 mm margin around the GTV is sufficient to achieve >100 Gy BED10 to the GTV merits further investigation. On the other hand, these results suggest that the dosing scheme of 54 Gy in 3 fractions always maintains a high BED10 (>100 Gy) to the both PTV and GTV even with simulated setup errors for a single‐isocenter/multiple lesions VMAT. Therefore, while there was underdosing of the PTV and GTV, it still resulted in a BED10 >100 Gy, implying that this dose and fractionation regimen is the regimen of choice. However, if the tumor is near critical structures and warrants five treatments, a higher dose per day (such as 11‐12 Gy for five fractions as seen in the RTOG 0813 trial) can be considered.

For many patients, remaining in the treatment position for long periods may be uncomfortable and result in intrafraction motion, causing the desire for faster yet effective treatment plans. Bissonnette *et al* demonstrated that spatial errors, although typically small in lung SBRT, could be larger with longer treatment times.^27^ A study by Hoogeman *et al* reported that intrafraction setup errors will increase linearly with treatment time, giving incentive to decrease the treatment time for single‐isocenter multi/lesions VMAT.^28^ Although this simulation study does not account for intrafraction setup errors, this consideration would increase uncertainty. Treating patients faster with a single‐isocenter VMAT plan could minimize intrafraction patient motion errors and improve patient comfort. Additionally, clinical follow‐up in a larger patient cohort is ongoing to see the effectiveness of this treatment approach.

Despite growing interest in single‐isocenter/multiple‐lesion VMAT lung SBRT treatments, difficulties due to daily patient setup errors have been described. When treating multiple lesions with a single‐isocenter VMAT plan, a physician has the task of lining up all the lesions on a daily CBCT images. It has been reported that boney anatomy cannot be used as a surrogate for soft tissue matching for lung SBRT treatment.^29^ A clinical study by Trager *et al* demonstrated that when two lesions share a same isocenter, approximately 30% of the time both lesions do not line up correctly in a single CBCT images.^30^ Thus, the physician is faced with a dilemma: what to do if the lesions do not line up correctly? The first option would be to align the lesions as best as possible, potentially “splitting‐the‐difference” if the differences are small and clinically acceptable. The second option would be to reposition the patient, repeat the CBCT scan and realign the lesions again. If, once again, the lesions do not line up properly the treating physician may need to abandon the treatment and either replan or try again. This can lead to delays in providing appropriate treatment, in addition to adding stress to the SBRT team and slowing down the clinic. Quan *et al* described the feasibility of treating ≥2 lesions with VMAT or intensity‐modulated radiosurgery (IMRS) and suggested that if all the lesions do not match up correctly on the daily pretreatment CBCT the only option is to abandon the SBRT treatment.^9^ Although aforementioned studies have shown the results of setup uncertainties in the case of a single‐lesion SBRT treated with an isocenter at the center of the target, the authors believe that this is the first study to report the results of patient misalignment for extracranial single‐isocenter/multilesion lung SBRT. Similarly, Clark *et al* demonstrated the dosimetric impact of rotational setup errors for single‐isocenter/multitarget VMAT SRS to multiple brain metastases using a “third party” software.^31^ It was reported that minimizing rotational setup errors was essential for adequate target coverage, even more so for small lesions in the brain and lesions far from the isocenter location. This study found that with even 2° rotations in all directions could result in an inadequate PTV target coverage with an average of 89.4 ± 10.6%, up to 100% in some cases, however, the study did not consider the translational setup errors. This current study does not use a “third party” software but rather a novel tool to preserve all treatment planning parameters including planning CT images and structure contours in Eclipse TPS, therefore introducing no additional sources of errors. This tool can be used for both extracranial multilesion and single‐lesion SBRT or intracranial SRS for multiple or single‐lesion treatment, as needed.

Although, the uses of PTV and GTV target(s) coverage are not ideal in this study. This is a limitation of this study, that is due to the clinical data available in our center. Utilizing the ITV coverage in all cases would be ideal for future investigation. That would account for intrafraction motion errors as well. However, as demonstrated, imperfect patient setup resulted in an unacceptable target coverage loss. To minimize this potential loss of target coverage while still allowing for a fast SBRT treatment of multiple lung lesions, ongoing research includes developing a novel method utilizing a single‐isocenter placed at patient’s midline and allowing for partial arcs to deliver dose to individual tumors. To minimize setup uncertainties, each plan can be reoptimized separately while sharing the same isocenter. This allows a SBRT plan to be created for each tumor, while allowing both tumors to be treated sequentially during the same session with soft tissue alinement one at a time while reducing chance of a geometric miss due to residual setup uncertainties. Placement of a single‐isocenter at patient’s mediastinum will avoid potential patient collisions and provide greater degree of noncoplanar arc geometry. It will eliminate the need of additional couch movements during CBCT imaging (couch centering is required for Varian Linac for lateral offsets of >5 cm, potentially introducing an additional source of error) and minimize the need for therapists to enter the treatment room for multiple couch positions.

## 5 CONCLUSION

A novel and simple method for demonstrating isocenter misalignment in six dimensions and the resulting dosimetric impact for single‐isocenter VMAT lung SBRT plans for two lesions has been presented. Clinically representative patient setup errors may result in large deviations (up to 72% loss) from planned target coverage. Smaller targets show the largest deviation from planned coverage including delivering <100 Gy BED10 to some targets. Small misalignments can result in substantial decrement in dose gradient and significantly increase the intermediate dose‐spillage. When treating small lesions synchronously with a single‐isocenter VMAT lung SBRT plan, alternative treatment planning strategies should be explored to minimize the likelihood of a geometric miss.

## CONFLICT OF INTEREST

The author have no other relevant conflict of interest to disclose.

## Author’s contributions

DP and LCC conceived the project. LCC and DP developed the algorithm, collected and analyzed the data. DP, RM, MB, and MR provided clinical expertise and supervision of the paper. LCC and DP drafted the manuscript and all coauthors revised and approved the final manuscript.
